# Further characterisation of transmissible spongiform encephalopathy phenotypes after inoculation of cattle with two temporally separated sources of sheep scrapie from Great Britain

**DOI:** 10.1186/s13104-015-1260-3

**Published:** 2015-07-24

**Authors:** Timm Konold, Romolo Nonno, John Spiropoulos, Melanie J Chaplin, Michael J Stack, Steve A C Hawkins, Saira Cawthraw, John W Wilesmith, Gerald A H Wells, Umberto Agrimi, Michele A Di Bari, Olivier Andréoletti, Juan C Espinosa, Patricia Aguilar-Calvo, Juan M Torres

**Affiliations:** Specialist Scientific Support Department, Animal and Plant Health Agency Weybridge, New Haw, Addlestone, Surrey KT15 3NB UK; Department of Veterinary Public Health and Food Safety, Istituto Superiore di Sanità (ISS), Viale Regina Elena 299, 00161 Rome, Italy; Pathology Department, Animal and Plant Health Agency Weybridge, New Haw, Addlestone, Surrey KT15 3NB UK; Prion Unit, Virology Department, Animal and Plant Health Agency Weybridge, New Haw, Addlestone, Surrey KT15 3NB UK; Formerly Epidemiology Department, Veterinary Laboratories Agency Weybridge, New Haw, Addlestone, Surrey KT15 3NB UK; Formerly Neuropathology, Veterinary Laboratories Agency Weybridge, New Haw, Addlestone, Surrey KT15 3NB UK; UMR INRA-ENVT 1225, Interactions Hôte Agent Pathogène, École Nationale Vétérinaire de Toulouse, 31076 Toulouse Cedex 3, France; Centro de Investigación en Sanidad Animal (CISA-INIA), Madrid, Spain

**Keywords:** Scrapie, Experimental challenge, Cattle, Bovine spongiform encephalopathy, BSE, Prion, L-type BSE, Western immunoblot, Bank vole, Transgenic mice

## Abstract

**Background:**

The infectious agent responsible for the bovine spongiform encephalopathy (BSE) epidemic in Great Britain is a transmissible spongiform encephalopathy (TSE) strain with uniform properties but the origin of this strain remains unknown. Based on the hypothesis that classical BSE may have been caused by a TSE strain present in sheep, cattle were inoculated intracerebrally with two different pools of brains from scrapie-affected sheep sourced prior to and during the BSE epidemic to investigate resulting disease phenotypes and characterise their causal agents by transmission to rodents.

**Results:**

As reported in 2006, intracerebral inoculation of cattle with pre-1975 and post-1990 scrapie brain pools produced two distinct disease phenotypes, which were unlike classical BSE. Subsequent to that report none of the remaining cattle, culled at 10 years post inoculation, developed a TSE. Retrospective Western immunoblot examination of the brains from TSE cases inoculated with the pre-1975 scrapie pool revealed a molecular profile similar to L-type BSE. The inoculation of transgenic mice expressing the bovine, ovine, porcine, murine or human prion protein gene and bank voles with brains from scrapie-affected cattle did not detect classical or atypical BSE strains but identified two previously characterised scrapie strains of sheep.

**Conclusions:**

Characterisation of the causal agents of disease resulting from exposure of cattle to naturally occurring scrapie agents sourced in Great Britain did not reveal evidence of classical or atypical BSE, but did identify two distinct previously recognised strains of scrapie. Although scrapie was still recognizable upon cattle passage there were irreconcilable discrepancies between the results of biological strain typing approaches and molecular profiling methods, suggesting that the latter may not be appropriate for the identification and differentiation of atypical, particularly L-type, BSE agents from cattle experimentally infected with a potential mixture of classical scrapie strains from sheep sources.

**Electronic supplementary material:**

The online version of this article (doi:10.1186/s13104-015-1260-3) contains supplementary material, which is available to authorized users.

## Background

Epidemiological studies indicated that the bovine spongiform encephalopathy (BSE) epidemic in the United Kingdom (UK) was caused by food-borne exposure of cattle to a transmissible spongiform encephalopathy (TSE) agent [1], but the origin of that agent remains unknown. As previously summarised [2], the initial characterisation of the agent isolated from cattle, and from several mammalian species to which BSE was subsequently transmitted, naturally and experimentally, concluded that it was a single, unique strain of TSE agent unlike that of previously identified strains of scrapie of sheep [3–5]. Nevertheless, sheep scrapie was considered a source of TSE agents to which cattle were potentially exposed via commercial feed. Notwithstanding the possible modification of agents resulting from commercial feed processing, a study was initiated in 1997 to assess the pathogenicity of scrapie agents for cattle. By inoculating cattle intracerebrally with two pools of brains from classical scrapie-affected sheep sourced in Great Britain (GB) before and during the BSE epidemic this study could potentially identify an endemic form of scrapie that was pathogenic for cattle or a BSE agent present in the sheep population. Interim results published in 2006 identified two different disease phenotypes, neither of which were consistent with the then recognised stereotypic phenotype of BSE in cattle and isolates from these did not have the strain typing characteristics of the BSE agent on transmission to wild-type mice [2]. The present manuscript describes updated findings of the cattle transmission study following cull of all cattle remaining at 10 year post inoculation and includes further characterisation of the disease occurring in recipients using newly available molecular diagnostic and biological strain typing techniques.

Subsequent to the initiation of the study, two different disease phenotypes of naturally occurring BSE were described, termed H-type [6] and bovine amyloidotic spongiform encephalopathy (BASE) or L-type BSE [7]. Later research suggested that these ‘atypical’ BSE forms arise spontaneously in cattle and may have been the origin of the agent responsible for the BSE epidemic, termed now ‘classical’ or ‘C-type’ BSE [8, 9]. To aid in the differentiation of these atypical forms of BSE from classical BSE, a new postmortem test protocol was proposed for the molecular discrimination of isolates [10]. It was these advances and the availability of new transmission models that were used to improve characterisation of isolates from the initial study. The transmission models included the use of bank voles, which are particularly susceptible to certain ovine scrapie strains [11], even from sources that are poorly or not transmissible to conventional and transgenic mice [12]. Transgenic mice expressing the ovine [13] or bovine prion protein (*PrP*) gene [14] were also used to facilitate transmissibility of ovine and bovine derived TSEs respectively.

## Methods

All procedures were carried out following ethical review in the authors’ respective institutions and in accordance with the European (European Community Council Directive 86/609/EEC) and the following national legislation: Home Office approval under the Animal (Scientific Procedures) Act 1986 and relevant project licences in the UK; Italian Ministry of Health authorisation according to Legislative Decree 116/92; agreement numbers 02-032-02 for animal care facilities, 92–189 for animal experimentation in France and the Committee on the Ethics of Animal Experiments of the INIA (Permit Numbers: M03043 and CEEA2O12/O24) approval in Spain. In each of the following sections the biological strain typing and WB profiling methods applied for phenotypic characterization are described in relation to the host models utilised.

### Transmission of scrapie to cattle

The methods of this study were presented in detail previously and Additional file 1 summarises the experimental design and outcomes [2].

Briefly, two groups of cattle were inoculated intracerebrally with brain homogenate from pathologically confirmed scrapie cases sourced prior to 1975 (ten cattle) and after 1990 (ten cattle). Both inocula were characterised by biochemical (Western immunoblot (WB) hybrid technique [15]) and biological (transmission in C57Bl, RIII and VM mice) approaches, which suggested that both contained classical scrapie isolates. Controls comprised five cattle inoculated intracerebrally with New Zealand-derived ovine brain homogenate [with no detectable disease-associated prion protein (PrP^Sc^) in the brain] and five cattle inoculated intracerebrally with saline solution. The study was terminated at 120 months post inoculation (mpi), and all remaining cattle (one from the pre-1975 group, three from the post-1990 group and controls) were euthanased with pentobarbitone. Pathological examinations were carried out as described previously [2]. The WB protocol used formerly was modified and applied retrospectively to previously positive samples. The modified protocol was based on the BioRad TESeE WB method (BioRad Laboratories, Marnes-La-Coquette, France) using mAb Sha31 (BioRad Laboratories) in place of the previously used mAb 6H4 (Prionics AG, Schlieren, Switzerland) and mAb P4 (Biopharm, Darmstadt, Germany) [16]. These mAbs target the PrP amino acid (aa) residues 156–163 (Sha31), aa 155–163 (6H4), and aa 97–112 (P4) of the sheep *PrP* sequence. In addition, mAb SAF84 (bovine aa sequence 175–180, kindly supplied by Dr. T Baron, AFSSA, France) was used because of its particular usefulness in identifying H-type BSE by specific downward molecular mass shift [17] and the potential to discriminate between CH1641-like scrapie and L-type BSE in ovine transgenic mice [18].

With this WB technique proteinase-resistant prion protein (PrP^res^) is detected as three protein bands that relate to diglycosylated, monoglycosylated and unglycosylated forms of the abnormal protein, and the migration as well as the relative intensity (expressed as glycoform ratio) of the protein bands of PrP^res^ enables differentiation of scrapie from BSE. Discrimination is also possible by parallel testing with the two specific mAbs: mAb Sha31 detects PrP^res^ in both cattle and sheep, while mAb P4 is more selective for scrapie PrP^res^ under the test conditions, as reported previously [15]. All positive samples were subjected to mild and stringent proteinase K (PK) digestion [10, 19] and the blotted PrP^res^ bands detected using mAbs Sha31, 6H4 and P4. Stringent digestion was undertaken with 500 μg/ml PK at pH 8.0, and mild digestion with 50 μg/ml PK at pH 6.5. The PK susceptibility ratio was obtained by comparing the optical density of the signal strengths of the PrP^res^ bands produced by mild and stringent digestion, which is >0.7 for C-type BSE and <0.6 for L-type BSE cases [10, 19].

Determination of the bovine *PrP* gene of the cattle was repeated for the purpose of this update to better determine potential genetic susceptibility factors influencing the outcome of transmissions and included examination of the promoter region and full open reading frame (ORF) from either blood (live animal) or brain (culled animal) according to methods described previously [20].

The original study design included strain characterisation only in wild-type mice but brain tissue was subsequently distributed to other research institutes to further characterise bovine passaged scrapie in additional rodent lines. This was carried out independent of the original study and selection of material was restricted by availability.

### Transmissions in bank voles

Bank voles (*Myodes glareolus*) were inoculated with brain tissue (parietal cortex) from steers P75-7 (inoculated with the pre-1975 pool) and P90-4 (inoculated with the post-1990 pool), both positive for PrP^Sc^ in brain. Both inocula were further tested by WB for presence of PrP^res^ as described previously using mAb SAF84 (Bertin Pharma, Montigny le Bretonneux, France) and mAb P4 (Biopharm, Milan, Italy) [21].

Each of two groups of 15 bank voles (homozygous for methionine at codon 109 of the *PrP* gene) were inoculated with 20 μl of brain homogenate (as 10% w/v in PBS) derived from each of the two steers. Inoculation procedure, clinical monitoring and euthanasia at terminal stage of disease were as described previously [12]. Brains from individual voles culled at terminal stage of disease were used for subsequent passages using the same protocol. Individual vole brains were used for strain typing by biochemical PrP^res^ characterisation and lesion profiling. Brains were examined by WB with mAb SAF84 targeting PrP aa residues 163–173 of the bank vole *PrP* sequence and 12B2 (CVI, Lelystad, Netherlands; PrP aa residues 89–93 of the sheep *PrP* sequence) [22], and lesion profiles were carried out by scoring vacuolar changes in nine grey matter areas of the brain on H&E stained sections [12].

The disease phenotypes observed in voles after transmission of P75-7 and P90-4 brain samples were compared with those previously derived from different scrapie sources, including natural ovine scrapie isolates SS-UK6 (10 brains) and SCR6 (single brain) [12] and the experimental CH1641 isolate (kindly provided by N. Hunter, Roslin Institute, University of Edinburgh, UK).

### Transmissions in transgenic mice

Inocula comprised the original two scrapie brain pools (pre-1975 and post-1990) and brain tissue (thalamus, with detectable PrP^Sc^) from the clinically affected steers P75-7 (as above) and P90-1 (inoculated with the post-1990 pool). Both of the original inocula and the brain of steer P90-1 had previously been inoculated into conventional mice (RIII, C57Bl and VM), resulting in successful transmission with lesion profile features uncharacteristic of BSE (pre-1975 pool), or transmissions with low attack rates, insufficient to establish a lesion profile in RIII mice (post-1990 pool and P90-1) [2]; see also Additional file 1 for a summary.

Inoculations were carried out in transgenic mouse lines expressing the *PrP* gene of various species as follows: tg338 mice (expressing the VRQ allele of the ovine *PrP* gene [23]), tg110 (expressing the bovine *PrP* gene [14]), tg001 (expressing the porcine *PrP* gene [24]), tga20 (over-expressing the murine *PrP* gene [25]) and tg340 (over-expressing the M_129_ allele of the human *PrP* gene [26]).

Groups of 6–12 mice were inoculated intracerebrally with 20 μl of either 2% homogenate of the original ovine brain pools or 10% homogenate of the bovine brain tissue (prepared in sterile 5% glucose). The former inoculum dilution was determined by restricted availability of source tissue. The procedures for inoculation, clinical monitoring and cull of affected mice were as described previously [9].

Disease in mice was confirmed according to previously published protocols for detection of PrP^res^ by WB with mAb Sha31 (BioRad Laboratories) [9] or paraffin embedded tissue blot (PET blot), which uses mAb Sha31 (BioRad Laboratories), followed by the application of an alkaline phosphatase coupled secondary antibody (Dako reference D0314—1/500 diluted, Dako France S.A.S, Les Ulis Cedex) and detection of enzymatic activity using NBT/BCIP substrate chromogen [27]. Brain homogenates from PrP^res^ positive mice, where available, were used for further passages. When, on primary passage, all mice of an inoculum group were negative for PrP^res^ a second passage of the pooled brain homogenates was carried out. For second passages, mice were inoculated intracerebrally with 20 μl of a 10% w/v brain homogenates (prepared in sterile 5% glucose).

Data were compared to those derived from mice inoculated with classical BSE bovine and ovine brain homogenates and with L-type and H-type BSE bovine brain homogenates, some of which were obtained from separate studies [9, 26, 28–31].

## Results and discussion

### Transmissions of scrapie to cattle

From the time of publication of interim results [2] to termination of the study, no additional cases of TSE, as confirmed by postmortem tests, were identified in the remaining cattle inoculated with the two scrapie pools. Thus, the attack rate remained at 9/10 in cattle inoculated with the pre-1975 pool and 7/10 in cattle inoculated with the post-1990 pool, with affected cattle presenting either with a clinical “nervous” syndrome (see Additional file 2: nervous form) or “dull” syndrome (see Additional file 3: dull form). Details of all cattle, with time and circumstances of death, clinical signs and diagnosis are presented in Table 1. Two of the three cattle (P90-8 and P90-9) receiving the post-1990 scrapie pool and surviving to termination, displayed some clinical signs similar to the previous seven pathologically confirmed cases within this inoculation group. Both were culled because they became recumbent and unable to rise. Whilst the finding of hypophosphataemia in one steer may have explained the clinical sign of difficulty rising, the clinical presentation of the other steer (see Additional file 4: unconfirmed suspect, showing steer P90-8 with difficulty rising and standing motionless at the side of the pen), which was reminiscent of a milder form of the dull syndrome, remains unexplained. We previously reported the occurrence of clinical signs suggestive of TSE in experimentally inoculated or naturally exposed farm animal species where PrP^Sc^ or PrP^res^ could not be detected on examination of the brain by postmortem tests [32–34] and cannot exclude a similar phenomenon in this steer. Inoculation of mice with brain tissue would be required to investigate whether this steer developed a prion disorder that could not be confirmed by current statutory TSE diagnostic tests.

**Table 1:** Animal details and outcome of cattle inoculated intracerebrally with two scrapie pools

Case	ORF	23 bp	12 bp	Death (mpi)	Reason for cull (clinical and/or pathological diagnosis)	TSE test result
Pre-1975 pool						
P75-1	6:6 N192 het	−/−	−/−	18	TSE suspect (dull syndrome)	Positive
P75-2	6:6 Q78 het	+/−	+/−	21	TSE suspect (dull syndrome)	Positive
P75-3	6:6 WT	−/−	−/−	24	TSE suspect (dull syndrome)	Positive
P75-4	6:6 N192 het	−/−	−/−	24	TSE suspect (dull syndrome)	Positive
P75-5	6:5 Q78 het	+/−	+/+	24	TSE suspect (dull syndrome)	Positive
P75-6	6:6 Q78 het	+/−	+/−	26	TSE suspect (dull syndrome)	Positive
P75-7	6:6 WT	−/−	−/−	29	TSE suspect (dull syndrome)	Positive
P75-8	6:6 WT	−/−	−/−	34	TSE suspect (dull syndrome)	Positive
P75-9	6:6 Q78 het	+/−	+/−	56	TSE suspect (dull syndrome)	Positive
P75-10	6:6 Q78 het	+/−	+/−	91	Muscle trauma	Negative
Post-1990 pool						
P90-1	6:6 N192 het	−/−	−/−	18	TSE suspect (dull syndrome)	Positive
P90-2	6:5 Q78 het	+/−	+/+	24	TSE suspect (nervous syndrome)	Positive
P90-3	6:6 Q78 het	+/−	+/−	25	TSE suspect (dull syndrome)	Positive
P90-4	6:5 Q78 het	+/−	+/+	30	TSE suspect (dull syndrome)	Positive
P90-5	6:6 Q78 hom	+/+	+/+	32	TSE suspect (nervous syndrome)	Positive
P90-6	6:6 Q78 het	+/−	+/−	35	TSE suspect (dull syndrome)	Positive
P90-7	6:6 WT	−/−	−/−	54	TSE suspect (dull syndrome)	Positive
P90-8	6:6 Q78 het	+/−	+/−	84	TSE suspect (difficulty rising, dullness)	Negative
P90-9	6:6 Q78 het	+/−	+/−	99	Difficulty rising, stiffness, hypophosphataemia	Negative
P90-10	6:6 Q78 het	+/−	+/−	120	End of study	Negative
Saline solution						
CSa-1	6:6 WT	−/−	−/−	60	Difficult to handle (cryptorchid)	Negative
CSa-2	6:6 Q78 hom	+/−	+/−	101	Stiffness, visual impairment (strabismus, exophthalmos)	Negative
CSa-3	6:6 Q78 het	+/−	+/−	103	Osteoarthrosis	Negative
CSa-4	6:5 WT	−/−	+/−	115	Vertebral fracture	Negative
CSa-5	6:6 Q78 het N192 het	+/−	+/−	120	End of study	Negative
Scrapie-free brain						
CB-1	6:6 Q78 het	+/−	+/−	82	Spastic syndrome, osteoarthrosis	Negative
CB-2	6:6 WT	−/−	−/−	82	Spastic syndrome, osteoarthrosis	Negative
CB-3	6:6 Q78 het	+/−	+/−	120	End of study	Negative
CB-4	6:6 Q78 het N192 het	+/−	+/−	120	End of study	Negative
CB-5	6:6 WT	−/−	−/−	120	End of study	Negative

*ORF* open reading frame of the bovine *PrP* gene detailing the number of N-terminal octapeptide repeats, the silent polymorphisms Q78 and N192, either homozygous (hom) or heterozygous (het) at position 78 and 192 of the ORF respectively compared to the wild type (WT); and the 23 and 12 bp indels (*−* deletion allele, *+* insertion allele) of the promoter *PrP gene*, *mpi* months post inoculation, rounded down to the nearest month.

**Fig. 1:**
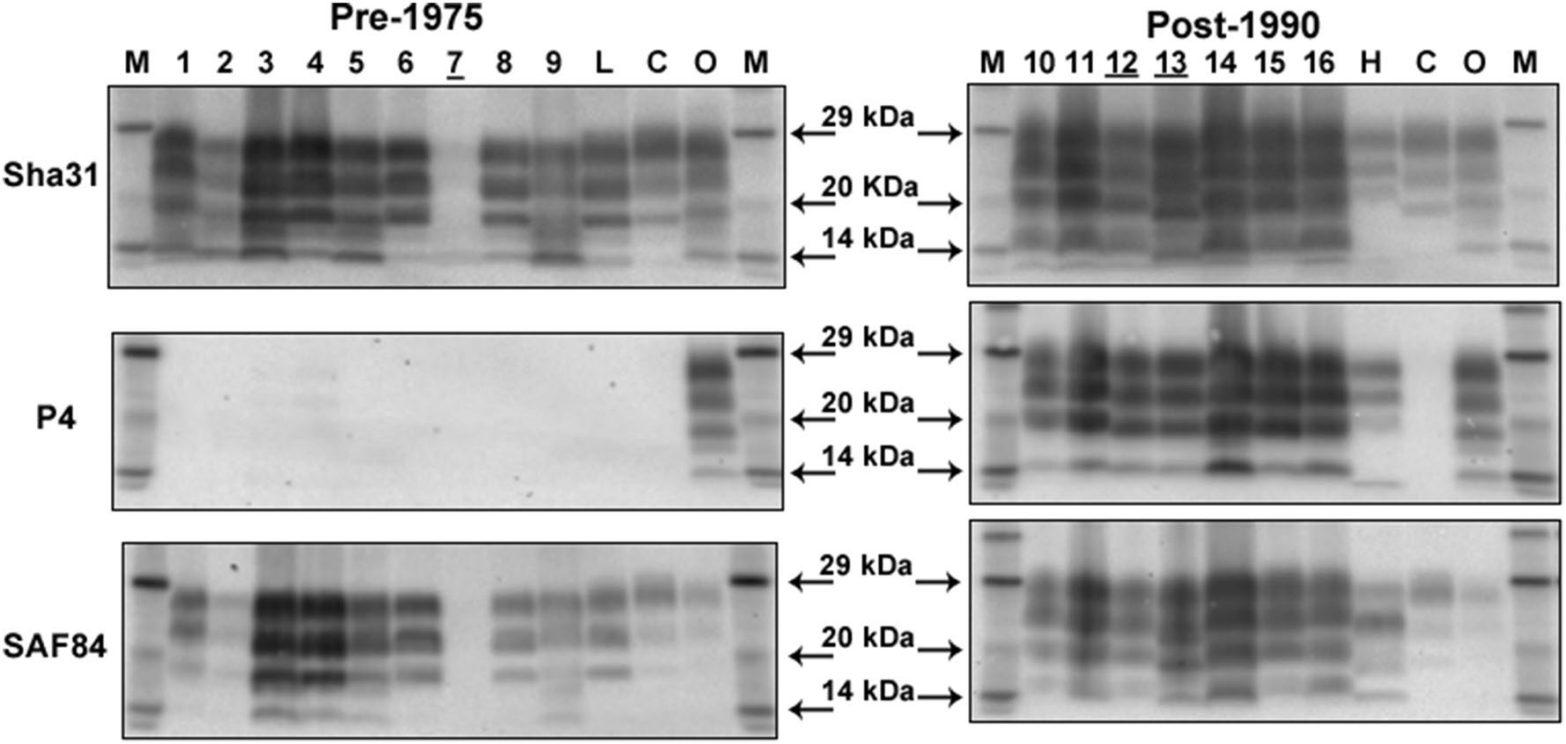
Discriminatory Western immunoblot of brain samples from cattle inoculated with the pre-1975 and post-1990 scrapie brain pools. *Lanes* *1*–*9* cattle inoculated intracerebrally with the pre-1975 scrapie pool: P75-1, P75-2, P75-3, P75-4, P75-5, P75-6, P75-7, P75-8 and P-75-9. *Lanes* *10*–*16* cattle inoculated intracerebrally with the post-1990 scrapie pool: P90-2, P90-3, P90-1, P90-4, P90-5, P90-6 and P90-7. *Lanes* *L*, *H*, *C*, *O* controls: L-type BSE, H-type BSE, classical BSE, ovine scrapie. *Lanes* *M* molecular mass marker. Animal P90-4, sample *lane 13*, was an outlier with a lower molecular mass of the unglycosylated band with mAbs Sha31 and SAF84 compared to the other samples previously tested with mAb 6H4. The sample of the other outlier P90-5 (determined previously by testing caudal medulla), sample *lane 14*, consisted here of rostral medulla and gave a molecular profile similar to the others of the group as observed in the original blot when both brain samples were tested [2]. The lane numbers of those cattle that provided the inocula for bank voles and mice are *underlined.*

**Fig. 2:**
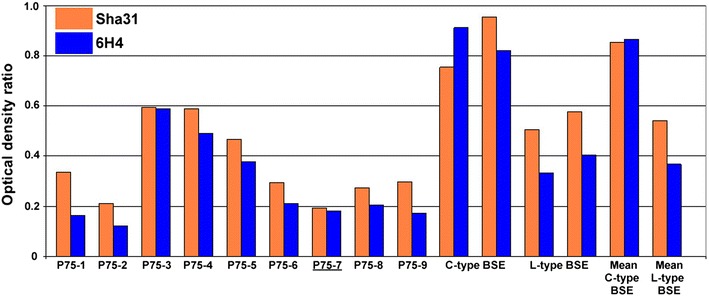
PK susceptibility ratio for the mild and stringent digestion conditions of samples from the pre-1975 scrapie pool compared to BSE. Controls comprise a UK classical BSE (C-type) sample (two analyses of the same sample and mean) and a UK L-type BSE sample (two analyses of the same sample and mean). Detection with mAbs Sha31 and 6H4. The case that provided the inoculum for bank voles and mice is *underlined.*

The brainstem samples of the nine positive cases inoculated with the pre-1975 pool, where the initial WB results resembled classical BSE, but with some differences regarding lower molecular mass migration and glycoform ratio, produced a WB profile with similarities to the L-type BSE control sample (see Fig. 1). On application of the PK susceptibility assay using mild and stringent conditions the susceptibility ratio for all cases was, like L-type BSE, below or close to the cut off level of 0.6, compared to the ratio for the classical BSE control of >0.7 (see Fig. 2).

The brain samples of the seven positive cattle inoculated with the post-1990 scrapie pool, the molecular profile of which previously resembled classical scrapie, maintained the classical scrapie profile with the WB protocol adapted for the detection of atypical BSE cases. They also exhibited variation in the molecular mass migration as reported previously [2]. After application of the PK susceptibility assay using mild and stringent conditions, the susceptibility ratio showed variation between the cases, with five being in the range 0.6–0.8, close to the cut off level of <0.6 for L- or H-type BSE, and two being susceptible to digestion at 0.4 and 0.5.

None of the samples from the pre-1975 or the post-1990 scrapie pools resembled an H-type BSE-like profile using mAbs Sha31 or P4 (no higher unglycosylated band) or showed the distinctive molecular mass downward shift and sharp band at 14 kDa, as illustrated by the H-type BSE control when SAF 84 was applied (see Fig. 1).

**Fig. 3:**
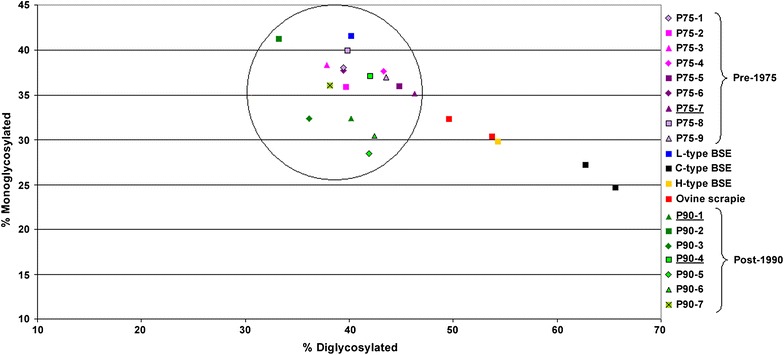
Scattergram showing the relative glycosylation quantity analysis of brain material from cattle inoculated with the pre-1975 and post-1990 scrapie pools compared to BSE and ovine scrapie controls. Detection with mAb Sha31. Controls comprised an ovine scrapie case, a classical BSE case (two analyses of the same sample each) and L-type and H-type BSE cases from the UK. The *black circle* indicates all 16 samples from the intracerebrally inoculated cattle clustering together around the L-type BSE control (*blue square*). The cases that provided the inocula for bank voles and mice are *underlined.*

Glycoform analysis of the di- versus monoglycosylated bands showed that the proportion of diglycosylated PrP^res^ was less than 50%, with all samples from both the pre-1975 and the post-1990 pools clustering with the L-type BSE control, whereas the classical BSE control sample showed a clear predominance of the diglycosylated bands giving more than 50% signal strength (see Fig. 3). Results using additional characterisation techniques for C- and L-type BSE [10, 19] were equivocal. Although the samples from the both groups of scrapie-inoculated cattle exhibited glycoform ratios more closely related to L-type BSE, previous experience has shown that ovine scrapie tends to have a more even distribution of the di- and monoglycosylated bands compared to C-type BSE (MJ Stack and MJ Chaplin, unpublished observation). Likewise, application of the PK susceptibility assay on a small number of scrapie samples has shown these samples can be close to the cut off point of L-type BSE (MJ Stack and MJ Chaplin, unpublished observation). Although these additional approaches have proven valuable in differentiating between bovine L- and C-type BSE [10, 19], they may not be reliable for differentiating WB profiles in cattle following infection from an ovine scrapie source. However, the results from the vole transmissions (see below) for the two brain pools suggest that they are similar or identical to scrapie strains already isolated from other European natural sheep scrapie cases.

We previously reported phenotype diversity in cattle inoculated with the post-1990 scrapie pool: two cattle [P90-4 (inoculated into bank voles) and P90-5, see Table 1] presented with a molecular profile different to that of the other cattle in the group (see Fig. 1, lane 13 for P90-4) which could not be explained by the number of octapeptide repeats in the bovine *PRNP*. Extended genotyping of the bovine *PrP* gene to include the promoter region and full ORF further confirmed that the *PrP* genotype of the steers was not responsible for the differences. Although the *PrP* gene polymorphism (ORF and promoter region) of animal P90-5 was not found in any other steer, the genotype of P90-4 was identical to that of P90-2 despite having a different molecular mass profile. The inoculation of cattle with a pool of scrapie brain material, containing possibly multiple strains, remains the most likely reason for the observed diversity in the molecular profiles as hypothesised previously [2]. Similarly, the *PrP* gene polymorphism did not appear to be responsible for the lack of transmission in some animals since the same polymorphism was found in inoculated cattle with or without PrP^Sc^ accumulation in the brain (see Table 1) and all scrapie-inoculated cattle with no evidence of PrP^Sc^ in the brain were heterozygous carriers of the 12 base pair (bp) deletion allele, which is associated with a higher risk of having BSE [35].

### Transmission in bank voles

**Fig. 4:**
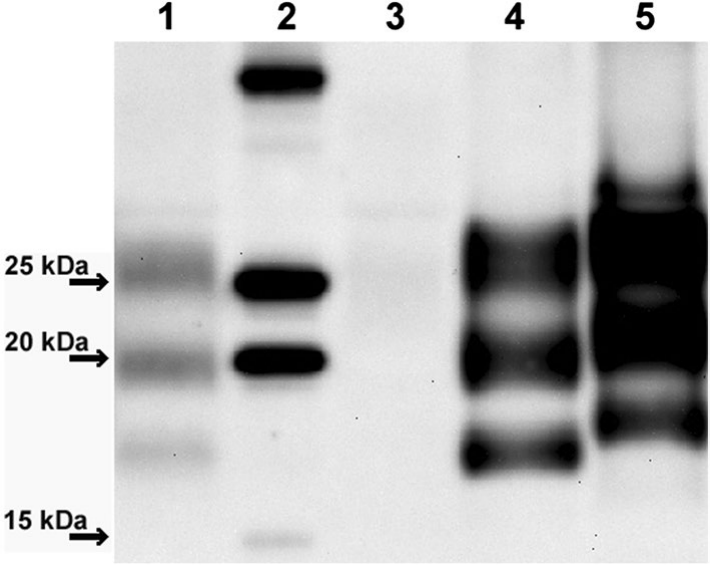
Western immunoblot of bovine brain samples used for transmission in bank voles and comparison with BASE and scrapie. (*1*) P75-7, (*2*) molecular mass marker, (*3*) P90-4, (*4*) BASE, (*5*) classical scrapie. Detection with mAb SAF84.

Prior to inoculation the bovine donor brain samples were subject to molecular analyses. Low levels of PrP^res^ were detectable by WB with mAb SAF84 in the inoculum from P75-7, whilst PrP^res^ was not detected in the inoculum from P90-4 (Fig. 4). The molecular profile of P75-7, in terms of both molecular mass and glycoform ratios showed some similarity to those of BASE in cattle, CH1641 and natural CH1641-like isolates in sheep. Accordingly, P75-7 was PrP^res^-negative with mAb P4 by discriminatory WB. Re-analysis after PrP^res^ concentration yielded identical results for the inoculum from P75-7 whilst very low levels of PrP^res^, which displayed a classical scrapie-like molecular profile (data not shown), were detected in the inoculum from P90-4. These findings were consistent with those made by separate WB analysis of the bovine brainstems (see above), which indicated that the molecular profile was maintained within different brain areas regardless of the choice of antibodies Sha31 or SAF84.

**Fig. 5:**
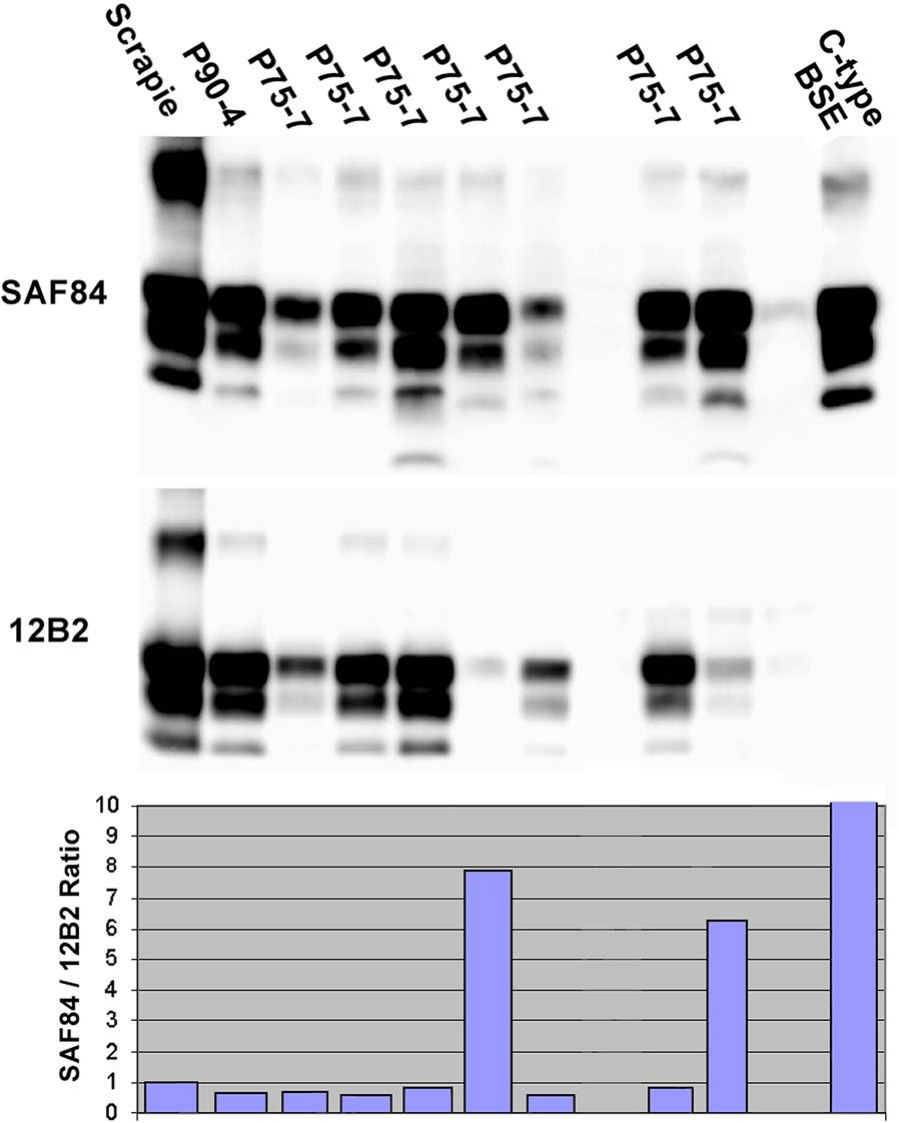
Western immunoblot and antibody signal ratio of vole brain samples after primary passage of bovine scrapie and comparison with vole-adapted classical scrapie and BSE. WB analysis of PrP^res^ in voles infected with P75-7 (primary passage) compared with a representative sample of P90-4 (primary passage), vole-adapted classical scrapie and BSE. Replica blots were developed with mAbs SAF84 (*upper panel*) and 12B2 (*lower panel*) to compare the antibody reactivity with each sample similar to the discriminative WB used for samples from small ruminants. The *graph* on the *bottom* shows the SAF84/12B2 signal ratio relative to the scrapie control. Values higher than 1 denote more C-terminal PK-cleavage and consequential loss of the epitope of 12B2.

On primary transmission in bank voles P75-7 gave a relatively long survival time (627 ± 72 days post inoculation, dpi) and a low attack rate (7/12). The PrP^res^ pattern in infected vole brains analysed by WB was not uniform (Fig. 5), with individual voles showing either a high molecular mass unglycosylated PrP^res^ fragment (n = 5, ~18 kDa, classical scrapie-like) or low molecular mass unglycosylated PrP^res^ fragment (n = 2, ~17 kDa, classical BSE-like). This partial similarity with classical BSE was confirmed by discriminatory WB, which showed that the 17 kDa PrP^res^ fragment in voles infected with P75-7 was poorly detected by mAb 12B2, the epitope of which (aa 93WGQGG97) is near the N-terminus of the PrP^res^ fragment (Fig. 5).

Second and third passages were made using donor voles displaying either the 18 or 17 kDa PrP^res^ fragment. The survival times and attack rates are displayed in Table 2.

**Table 2:** Survival times and attack rates of bank voles after inoculation with brains from P75-7 and P90-4 and comparison with other isolates

Inoculum	Survival time (attack rate) on primary transmission	Survival time (attack rate) on second passage	Survival time (attack rate) on third passage
P75-7	627 ± 72 (7/12)	18K 144 ± 7 (7/7)	18K 122 ± 5 (10/10)
		17K 145 ± 7 (14/14)	17K 111 ± 15 (6/6)
P90-4	382 ± 159 (11/15)	100 ± 5 (8/8)	95 ± 5 (7/7)
CH1641		18K 147 ± 11 (8/8)	18K 139 ± 9 (13/13)
		17K 112 ± 8 (10/10)	17K 119 ± 6 (10/10)
SCR6	197 ± 19 (18/18) [12]	98 ± 4 (11/11) [12]	94 ± 5 (11/11)
SS-UK6	175 ± 18 (22/22) [12]	96 ± 4 (11/11) [12]	85 ± 4 (8/8)

Survival times are expressed in days post inoculation with standard error of the mean.

K denotes the molecular masses (in kDa) of the unglycosylated PrP^res^ band determined by WB.

Overall, the transmission pattern observed with P75-7 had some similarities to that observed after transmission of CH1641 and CH1641-like natural sources in voles, which in previous experiments showed long incubation time and the presence of either 18 or 17 kDa PrP^res^ fragments after primary transmission (U Agrimi and R Nonno, unpublished observations). Also, the vole-adapted “sub-strains” derived from CH1641 gave survival times of ~110 dpi for the 17K sub-strain and of ~140 dpi for the 18K sub-strain (Table 2). These results differ from those obtained after transmission of L-type BSE (survival times ~400 dpi on second passage [36]) and classical BSE (survival times 483 ± 85 dpi on second passage [37]) in voles.

**Fig. 6:**
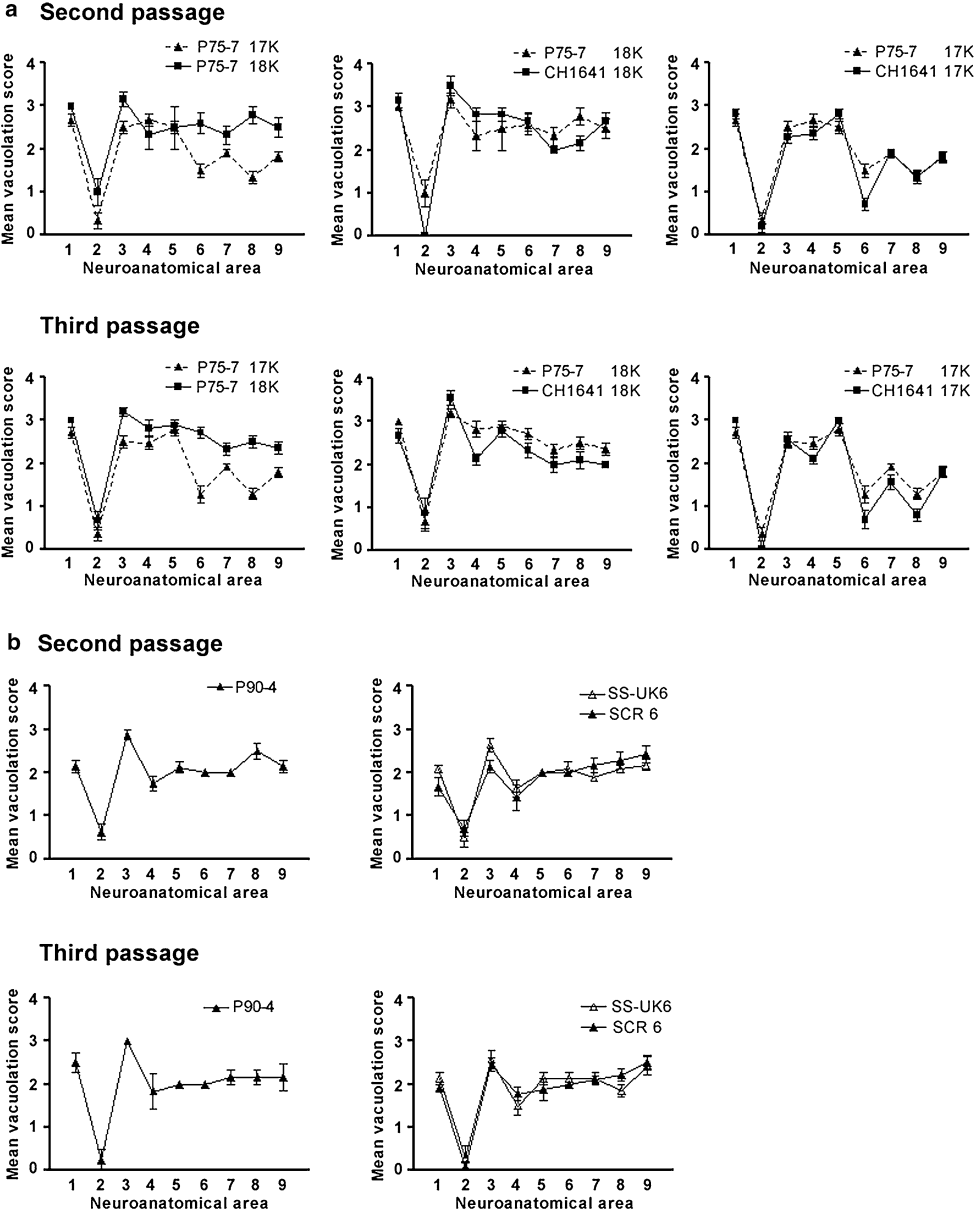
Lesion profiles of vole-adapted bovine scrapie at second and third passages and comparisons with other isolates. **a** Lesion profiles of vole-adapted P75-7 at second and third passages in voles in comparison with the two ‘sub-strains’ (17 and 18K) of vole-adapted CH1641. **b** Lesion profiles of vole-adapted P90-4 at second and third passages in voles in comparison with two natural scrapie isolates from sheep (SS-UK6 and SCR6). Scored neuroanatomical areas: *1* medulla, *2* cerebellum, *3* superior colliculus, *4* hypothalamus, *5* thalamus, *6* hippocampus, *7* septum, *8* retrosplenial and adjacent motor cortex, *9* cingulate and adjacent motor cortex.

Lesion profiles of the two vole-adapted sub-strains derived from P75-7 were slightly different, with the 18K sub-strain inducing more pronounced spongiform degeneration in the hippocampus and cerebral cortex compared to the 17K sub-strain (Fig. 6). These lesion and molecular profiles were again very similar to those obtained with CH1641 (Fig. 6). Although the P75-7 inoculum did not transmit to wild-type mice, brains from other steers inoculated with the pre-1975 scrapie pool transmitted to wild-type mice (RIII, C57Bl and VM), with a high attack rate in RIII mice [2], whereas CH1641 does not transmit to wild-type mice. Natural CH1641-like scrapie sources, however, produced a TSE in C57Bl mice [38], but the resulting lesion profile was different to that obtained from inoculation of C57Bl mice with brain from one pre-1975 inoculated steer (T Konold, unpublished observation). We cannot exclude the possible existence of several agent strains in the bovine brain, particularly as the inoculum for cattle was a pool of scrapie brains, which may have been selected variably in each of the species and forms of host models used. Similarly, minor variables in sampling for different techniques and studies may have resulted in testing of material with differing agent content.

The inoculum P90-4 also produced a long survival time (382 ± 159 dpi) and an attack rate of 11/15 although, based on the low level of PrP^res^ detection in the WB, this may have been a result of the possibly lower infectious titre of the inoculum. All affected vole brains had a classical scrapie-like pattern by WB (Fig. 5).

Second and third passages gave short and consistent survival times (100 ± 5 and 95 ± 5 dpi, respectively).

Lesion profiles of vole-adapted P90-4 were different from those observed in P75-7 (Fig. 6). The survival time after adaptation and the lesion profile observed with P90-4 were very similar to those observed in previous experiments after transmission in voles of several ARQ/ARQ natural scrapie sources, including SCR6 that contributed to the post-1990 pool and others from the same UK flock of Suffolk sheep with endemic scrapie [12]; see the lesion profiles displayed in Fig. 6b for comparison. All these isolates belong to a category provisionally called “It93”, so called because to date, all scrapie isolates from Italy and some ARQ/ARQ isolates from the UK have shown the same biological features (U Agrimi and R Nonno, unpublished observation).

In summary the findings in bank voles suggest that the prion strains isolated from the cattle inoculated with the pre-1975 and the post-1990 scrapie brain pools were different and distinct from classical BSE and L-type BSE, but similar, or identical, to scrapie strains previously isolated from European natural sheep scrapie cases.

### Transmissions in transgenic mice

Table 3 gives details of the transmissions in transgenic mice inoculated with the pre-1975 and post-1990 scrapie brain pools and the brain tissue from each of two cases of scrapie transmissions in cattle, sourced from separate inocula groups.

**Table 3:** Mean survival times and attack rate in transgenic mice inoculated with two ovine scrapie pools, two single case sources of bovine scrapie and single case sources of classical and atypical BSE on primary and second passage

Inocula	Post-1990 scrapie pool	P90-1	Pre-1975 scrapie pool	P75-7	Bovine C-type BSE	Ovine C-type BSE	H-type BSE	L-type BSE
*BoPrP*-tg110								
1st passage	643 (1/5) 19 + 21K	173 ± 3 (6/6) 21K	457 ± 60 (3/6) 19K	203 ± 5 (6/6) 19K	295 ± 12 (6/6) BSE like^a^	234 ± 5 (6/6) BSE like	292 ± 5 (6/6) H-type^b^	207 ± 7 (6/6) L-type
2nd passage	282 ± 6 (6/6) 19K	190 ± 16 (6/6) 21K	191 ± 4 (6/6) 19K	200 ± 9 (6/6) 19K	265 ± 35 (6/6) BSE like	234 ± 3 (6/6) BSE like	296 ± 7 (6/6) H-type	199 ± 1 (6/6) L-type
*PoPrP*-tg001								
1st passage	>650 (0/6)	>650 (0/6)	>650 (0/6)	>650 (0/6)	498 ± 9^c^ (2/12)	458 ± 11^c^ (15/15)	>650 (0/6)^b^	>650 (0/6)
2nd passage	>650 (0/6)	>650 (0/6)	>650 (0/6)	>650 (0/6)	198 ± 6^c^ (15/15)	162 ± 4^c^ (13/13)	>650 (0/6)^b^	>650 (0/6)
*MuPrP*-tga20								
1st passage	571 ± 31 (3/6) 21K	440 ± 3 (5/6)	441 ± 67 (6/6) 21K	480 ± 13 (6/6)	473 ± 24 (6/6) BSE like	450 ± 48 (6/6) BSE like	ND	ND
2nd passage	159 ± 2 (6/6) 21K	408 ± 33 (3/6)	146 ± 41 (3/3) 21K	ND	147 ± 3 (6/6) BSE like	117 ± 3 (6/6) BSE like	ND	ND
*HuPrP*-tg340								
1st passage	ND	>650 (0/5)	ND	>650 (0/6)	>700 (1/12) BSE like	615 ± 84 (4/6) BSE like^d^	>700 (0/6)^b^	629 ± 35 (5/5) L-type
2nd passage	ND	>650 (0/5)	ND	>650 (0/6)	690 ± 35 (5/6) BSE like	564 ± 39 (5/5) BSE like	>700 (0/6)^b^	684 ± 45 (4/4) L-type
*OvPrP*-tg338								
1st passage	480 ± 19 (6/6) 21K	>638 (0/6)	69 ± 1 (6/6) 21K	148 ± 2 (5/6) 19K	704 ± 36 (6/7) BSE like^f^	560 ± 60 (5/5) BSE-like^f^	595 ± 18 (8/8) H-type^g^	432 ± 19 (6/6) BSE-like^f^
2nd passage	ND	545 (1/5)^e^ 19K	ND	ND	ND	178 ± 2 (4/4) BSE-like^f^	319 ± 10 (6/6) H-type^g^	141 ± 2 (7/7) BSE-like^f^

Survival times are displayed in days with standard error of the mean. K denotes the molecular masses (in kDa) of the unglycosylated PrP^res^ band determined by WB.

*ND* not done.

^a^Inoculum from clinical BSE suspect, 8 year-old Holstein-Friesian cow, UK.

^b^Inoculum from 03-2095, clinically healthy ≥8 year-old cow, France [9, 44].

^c^Previously published data from UK and French cases: C-type BSE (BSE2, 8 year-old Hereford crossbred cow, clinical suspect), ovine BSE (pool from 7 *ARQ/ARQ* sheep intracerebrally infected with brainstem from a naturally affected BSE cow in France) [31].

^d^Brain pool of *ARQ/ARQ* sheep inoculated with brain from a naturally infected BSE cow [26].

^e^One additional inoculated mouse died at 535 days but no TSE diagnosis was possible.

^f^Previously published data from French cases: Ovine BSE (case ARQ1), C-type BSE (case 3), L-type BSE (case 7) [29].

^g^Previously published data from French case 2 [30].

Neither of the ovine scrapie pools or inocula from P75-7 or P90-1 transmitted to tg001 mice on first or second passage (Table 3). Lack of transmission of different classical scrapie isolates in tg001 mice has been previously described [31]. It appears therefore that these mice are also resistant to infection with scrapie sourced from a bovine host, as they are to challenge with L-type BSE, and only susceptible to classical bovine and ovine BSE.

Although both original scrapie pools transmitted to tg338 mice, survival times were almost seven times shorter in mice inoculated with the pre-1975 scrapie pool. As both inocula produced disease in cattle with similar survival time ranges, it seems unlikely that this finding is due to a lower infectious titre in the post-1990 scrapie pool. Short survival periods are observed in tg338 mice (a *VRQ* PrP transgenic mouse line) inoculated with *VRQ/VRQ* scrapie sheep isolates, whereas longer survival times have been observed in tg338 mice inoculated with *ARQ/ARQ* scrapie sheep isolates [39]. Both pools contained brains from *VRQ/VRQ*, *ARQ/VRQ* and *ARQ/ARQ* sheep in similar proportions (J Foster, personal communication). It is more likely that the post-1990 brain pool contained isolates which have been shown not to propagate in wild-type mice but transmit to tg338 mice with similar long incubation periods regardless of genotype of the sheep (*VRQ/VRQ* or *ARQ/ARQ*) source [40]. In fact, the post-1990 scrapie pool did transmit poorly to wild-type mice [2], and historical transmission studies using some of the individual sheep brains that made up the pool showed that one isolate (SCR 6) did not transmit or had a low transmission rate in wild-type mice whilst others (SCR 4, 9-11) transmitted well ([41]; J Foster, personal communication).

The survival times in tg338 mice inoculated with the inoculum from P75-7 were more than three times shorter than with inocula from ovine or bovine BSE sources. By contrast, the inoculum from P90-1 failed to transmit at primary passage but transmitted weakly on second passage; a phenomenon, which has not previously been documented for any isolate from naturally infected cattle with TSEs in this mouse line.

**Fig. 7:**
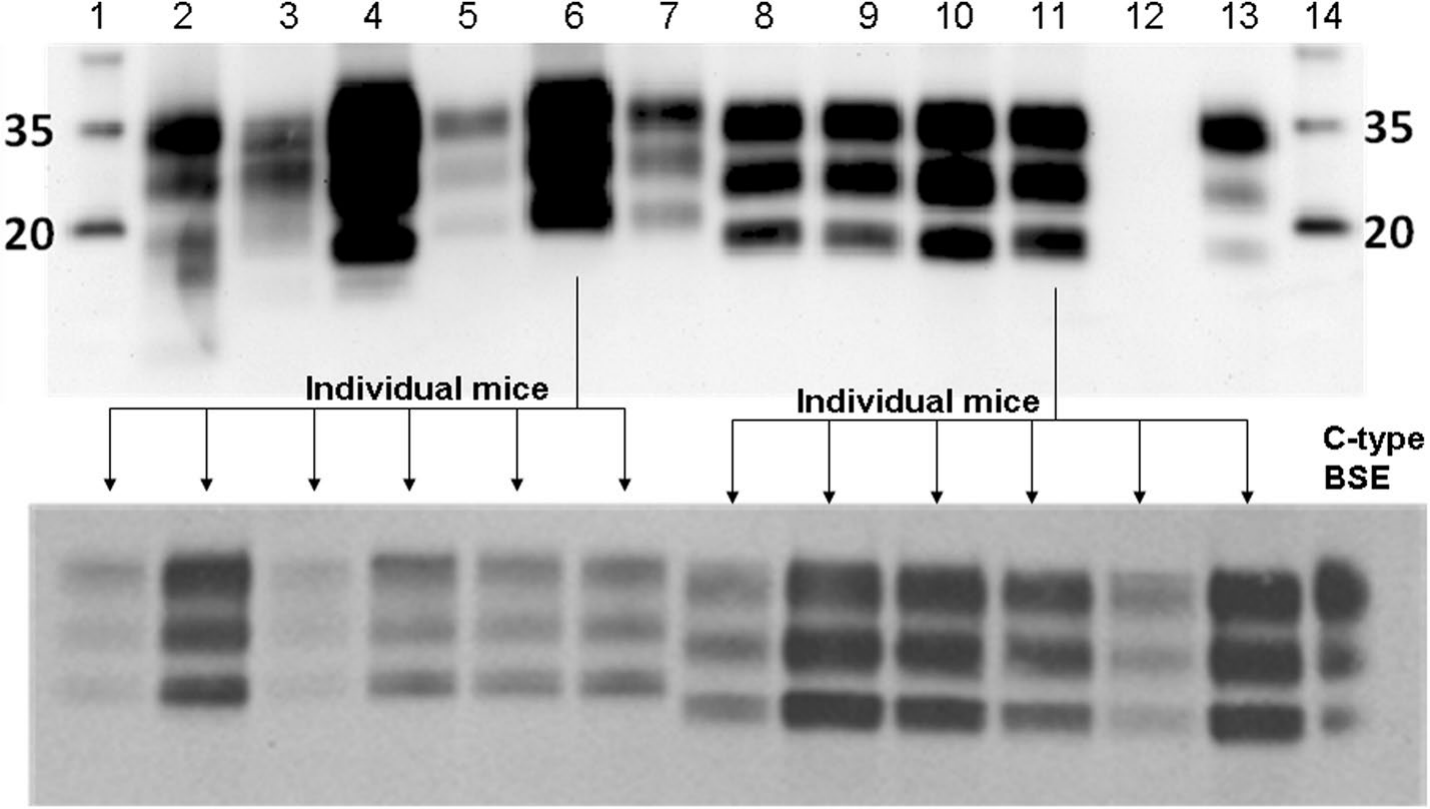
Western immunoblot profiles of tg110 mice inoculated with original scrapie brain pools and bovine scrapie sources P90-1 and P75-7. *Lanes* on *top blot*. *1* and *14* Molecular mass marker (kDa). *2* Post-1990 scrapie brain pool. *3* Post-1990 scrapie brain pool in Tg110 mice (first passage). *4* Post-1990 scrapie brain pool in Tg110 mice (second passage). *5* P90-1. *6* P90-1 in Tg110 mice (first passage). *7* Pre-1975 scrapie brain pool. *8* Pre-1975 scrapie brain pool in Tg110 mice (first passage). *9* Pre-1975 scrapie brain pool in Tg110 mice (second passage). *10* P75-7. *11* P75-7 in Tg110 mice (first passage). *12* Negative control (brain from non-inoculated Tg110 mice). *13* Positive control (brain from tg110 mice inoculated with classical BSE: case VLA-PG817/00). Antibody: Sha31. The molecular profile of the original post-1990 scrapie brain pool is maintained after passage in Tg110 mice (*lanes 2*–*4*) but does not resemble the profile of P90-1 or the mice inoculated with P90-1 brain (*lanes* *5*, *6*). By contrast, the profile of the original pre-1975 brain pool (*lane 7*) differed from the profile obtained from inoculated Tg110 mice (*lanes* *8*, *9*), P75-7 (*lane 10*) and the mice inoculated with P75-7 brain, which were all similar. The profiles obtained in individual mice after inoculation with the steers’ brains were identical within each inoculation group (*bottom blot*).

None of the WB profiles obtained after passage in tg110 mice resembled classical BSE. The profile in mice inoculated with the pre-1975 pool and P75-7 inoculum showed an unglycosylated band of 19 kDa, which was maintained after passage in all tg110 inoculated with this inoculum (see Fig. 7). This profile was reminiscent of CH1641, supported by the results in bank voles, which suggests that CH1641 was present in the pre-1975 scrapie pool, but this profile also resembled L-type BSE, like the WB profile of brain from steer P75-7, which provided the inoculum. A similar, L-type BSE-like WB profile has also been observed in tg110 mice inoculated with numerous non-CH1641 sheep and goat scrapie isolates from different European countries including France, UK, and Spain ([42]; JM Torres, unpublished data). Despite its resemblance to L-type BSE, the lesion profile and PrP^Sc^ distribution in the brain of tg110 mice was different to L-type BSE and as reported for CH1641 [43]. Another characteristic that distinguishes this isolate from L-type BSE was the lack of transmission in human-*PrP* transgenic mice (tg340) (Table 3). Furthermore, interpretation of the persistence of this molecular profile is also made difficult by the fact that the BASE or L-type BSE strain has been shown to convert into a classical BSE strain upon second passage in wild-type mice [8], although it maintained the L-type BSE-like profile in mice expressing the bovine *PrP* gene (Tg540 mouse line) [29]. Indeed, the majority of classical BSE cases that have been observed and examined with the necessary molecular detail were most likely the result of recycling of an agent within the cattle population, via meat and bone meal [45].

The WB profile obtained after inoculation of tg110 mice with the inoculum from P90-1 gave an unglycosylated 21 kDa band that has also been observed in tg110 mice inoculated with some sources of classical scrapie (JM Torres, unpublished data). A WB profile with an unglycosylated band of 19 kDa (L-type BSE-like) was also obtained after inoculation of tg110 mice with the post-1990 scrapie brain pool but the profiles obtained from the brain of steer P90-1 and tg110 mice inoculated with the inoculum from P90-1 were different (unglycosylated band of 21 kDa, in Fig. 7). It is not known whether the different infectious dose of the inoculum (1 ml of 10% in cattle versus 20 µl of 2% homogenate in mice), which is reflected in the attack rate (7/10 in cattle versus 1/5 in mice), contributed in any respect to this finding.

## Conclusions

Two different disease phenotypes were produced after intracerebral inoculation of cattle with scrapie brain pools sourced pre-1975 and post-1990 in GB, which were not readily explained by any differences in PrP genotype of the cattle. Based on pathological and molecular characteristics and biological characterisation in bank voles and transgenic mice there was no clear evidence of an agent derived from the cattle resembling classical or atypical forms of BSE. Transmissions in bank voles identified previously isolated scrapie strains and some similarities to the experimental isolate CH1641. Contrary to the transmission results in rodents, the results for the molecular techniques, which have been adopted for the detection of atypical BSE cases, suggest that they may not be appropriate for differentiating WB profiles in cattle following infection from an ovine scrapie source.

## Additional files

**Additional file 1: Summary of original experiment**. This document gives an overview of the inoculations carried out in cattle and wild-type mice with references to the animal numbers in the original, published study [2].
**Additional file 2: Nervous syndrome**. Steer P90-2 presenting with the nervous syndrome at 24 months after intracerebral inoculation with the post-1990 scrapie pool; TSE confirmed by postmortem tests. This steer displays an abnormal rising behaviour (attempt to rise on its fore limbs first when disturbed by a pen mate). Examination in a crush reveals over-reactivity to visual (menace response testing) and tactile facial stimuli (testing of sensation by touch with artery forceps) as well as tremor of the shoulder muscles. It is reluctant to walk towards the end of the corridor, seemingly apprehensive of the drain cover and groove on the floor, which it does not want to cross. There is mild hind limb ataxia as the steer walks away. Back in the pen, the steer is apprehensive of the approaching hand, tossing its head and wrinkling its nose before backing off, unlike the other steers in the pen, which appear curious.
**Additional file 3: Dull syndrome**. Steer P75-1 presenting with the dull syndrome at 18 months after intracerebral inoculation with the pre-1975 scrapie pool; TSE confirmed by postmortem tests. Initially, the clip shows two events of difficulty rising (the steer is briefly marked by an orange arrow to distinguish it from one of its pen mates). Dull demeanour is displayed on three separate occasions on the same day: the steer stands with its head between the wall and ladder in the pen, also temporarily resting its head on the ladder, for approximately two minutes (indicated by the counter on the top left corner of the clip). It also stands motionless with its head in the corner of the pen or in the gap between the wall and the pen divider. Loss of balance is evident during a neurological examination carried out two days later. The signs were suggestive of a diffuse or multifocal brain disease.
**Additional file 4: Unconfirmed suspect**. This clip shows steer P90-8 at 82–83 months after intracerebral inoculation with the post-1990 scrapie pool; TSE not confirmed by postmortem tests. Closed circuit television (time lapse VHS video recording in black and white, two cameras per pen marked 1 and 2) shows this animal (marked briefly with an orange circle or arrow to distinguish it from its pen mate) having difficulty rising (rocking several times, before rising on its fore limbs) and here shown on two occasions on separate days. After one of these episodes it walks along the pen divider and stands motionless with its head in the gap between the wall and the pen divider for almost two minutes (indicated by the counter on the right bottom corner of the clip), as seen in previous cases displaying the “dull” syndrome. The steer was culled one month later when it was found recumbent and unable to get up unaided.
