# Supplementary material for: Further characterisation of transmissible spongiform encephalopathy phenotypes after inoculation of cattle with two temporally separated sources of sheep scrapie from Great Britain
Source: BMC Res Notes. 2015 Jul 24;8:312. doi: 10.1186/s13104-015-1260-3 (PMC4618938; doi:10.1186/s13104-015-1260-3)
Supplement: Supplementary file 1 — Additional file 1: Summary of original experiment. This document gives an overview of the inoculations carried out in cattle and wild-type mice with references to the animal numbers in the original, published study [2]. [file 13104_2015_1260_MOESM1_ESM.pdf]

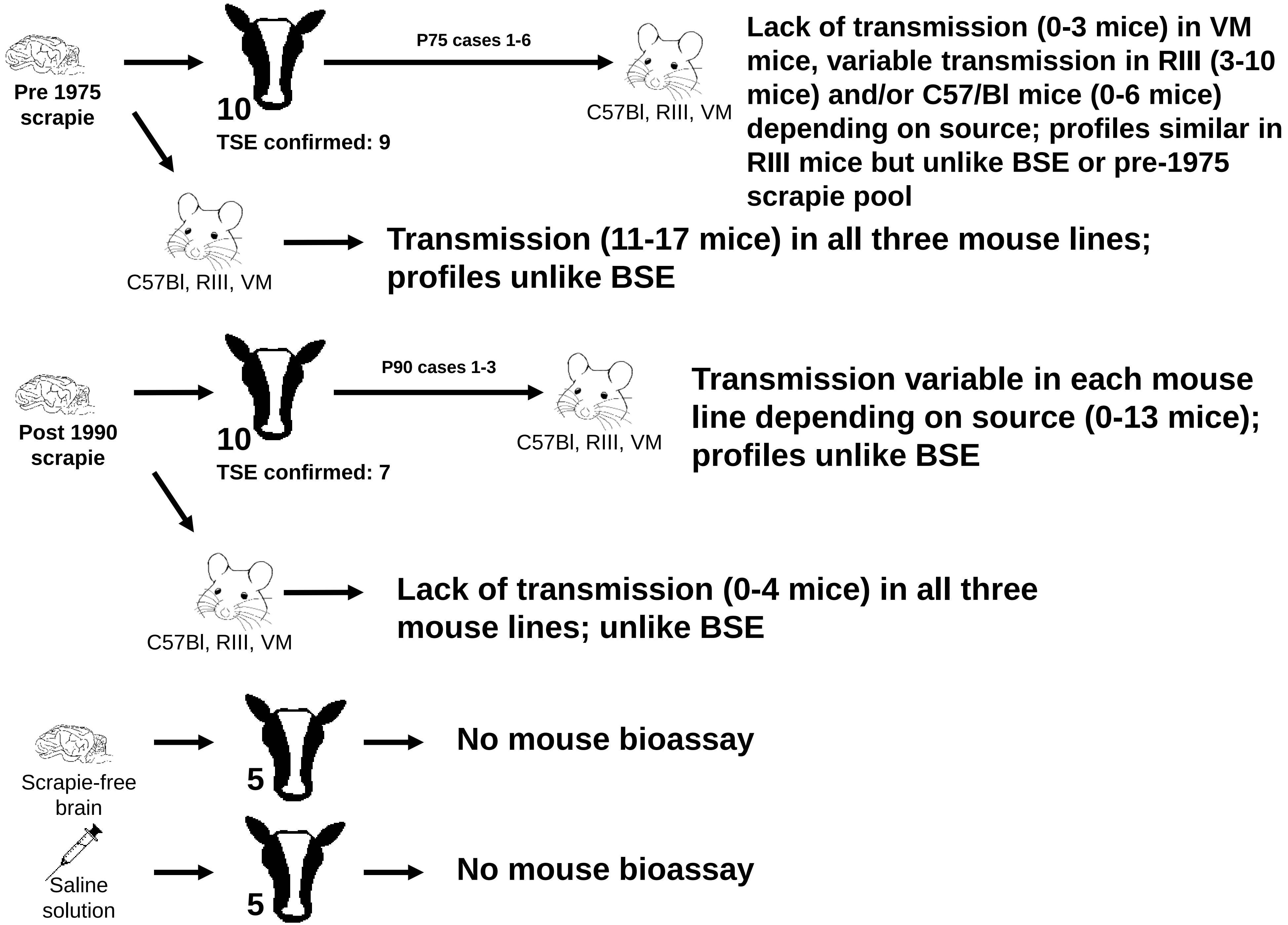

Pre 1975
scrapie
10
TSE confirmed: 9
P75 cases 1-6
C57Bl, RIII, VM
Lack of transmission (0-3 mice) in VM mice, variable transmission in RIII (3-10 mice) and/or C57/Bl mice (0-6 mice) depending on source; profiles similar in RIII mice but unlike BSE or pre-1975 scrapie pool
C57Bl, RIII, VM
Transmission (11-17 mice) in all three mouse lines; profiles unlike BSE
Post 1990
scrapie
10
TSE confirmed: 7
P90 cases 1-3
C57Bl, RIII, VM
Transmission variable in each mouse line depending on source (0-13 mice); profiles unlike BSE
C57Bl, RIII, VM
Lack of transmission (0-4 mice) in all three mouse lines; unlike BSE
Scrapie-free
brain
5
No mouse bioassay
Saline
solution
5
No mouse bioassay

Animal Reference Numbers

Inoculum	Case No	Animal ID
Pre 1975 pool	P75-1	119/01
	P75-2	609/01
	P75-3	743/01
	P75-4	749/01
	P75-5	959/01
	P75-6	1189/01
	P75-7	45/02
	P75-8	754/02
	P75-9	166/04
	P75-10	49/07
Post 1990 pool	P90-1	116/01
	P90-2	920/01
	P90-3	1063/01
	P90-4	152/02
	P90-5	512/02
	P90-6	1215/02
	P90-7	96/04
	P90-8	544/06
	P90-9	903/07
	P90-10	487/09
Saline solution	CSa-1	504/04
	CSa-2	919/07
	CSa-3	1071/07
	CSa-4	1477/08
	CSa-5	361/09
Scrapie-free brain	CB-1	60/07
	CB-2	84/07
	CB-3	40/10
	CB-4	41/10
	CB-5	42/10
